# Role of cervical ultrasonography in primary hyperparathyroidism

**DOI:** 10.4103/0971-3026.43846

**Published:** 2008-11

**Authors:** Feroze Shaheen, Nisar Chowdry, Tariq Gojwari, Arshad Iqbal Wani, Showkat Khan

**Affiliations:** Department of Radiodiagnosis and Imaging, SK Institute of Medical Sciences, Kashmir - 190 011, India; 1 Department of Surgery, SK Institute of Medical Sciences, Kashmir - 190 011, India; 2 Department of Endocrinology, SK Institute of Medical Sciences, Kashmir - 190 011, India; 3 Department of Nuclear Medicine, SK Institute of Medical Sciences, Kashmir - 190 011, India

**Keywords:** Primary hyperparathyroidism, Tc-sestamibi scan, ultrasonography

## Abstract

**Aim:**

To evaluate the role of USG in the preoperative localization of parathyroid adenomas in patients with symptomatic hyperparathyroidism and to compare its usefulness with that of scintigraphy scan and postoperative findings.

**Material and methods:**

Twenty-five patients with symptomatic primary hyperparathyroidism were subjected to USG of the neck and nuclear scintigraphy, followed by surgery. The results were independently analyzed and compared with per-operative findings.

**Results:**

The 25 patients had a total of 28 abnormal glands: 22 solitary adenomas, and 6 multiple adenomas (two each in three patients). USG detected 20 out of 22 solitary adenomas and three out of six multiple adenomas. USG missed five abnormal glands, two of which were in the neck and three in the mediastinum. Scintigraphy was positive in 26 abnormal glands, out of which 22 were single and four were multiple. Two abnormal glands were missed: one in the neck and one in the mediastinum.

**Conclusion:**

As limited neck dissection for primary hyperparathyroidism becomes increasingly popular, USG has been found to be a sensitive, specific, and easily available noninvasive investigation for parathyroid localization. It can be easily offered to patients as a method for preoperative localization prior to limited parathyroid surgery outside tertiary care settings.

Primary hyperparathyroidism (PHPT) is caused by solitary parathyroid tumors in nearly 85% of cases. Other causes are hyperplasia: 12–15%; multiple adenomas: 2–3%; and carcinoma: <1%.[1,2] The traditional surgical method of bilateral neck exploration under general anesthesia, although successful in 95% of patients, entails increased time, cost, and postoperative morbidity.[3] The recent emergence of surgical approaches like directed parathyroid exploration,[4] minimally invasive parathyroidectomy under local anesthesia,[5] and endoscopic parathyroidectomy with a small neck incision have been made possible by accurate preoperative imaging techniques.[6] Out of the many methods of localization, high-resolution USG and nuclear scintigraphy are the most commonly used. USG has advantages over scintigraphy in that it is easily available, more convenient, and less expensive. Given the fact that increasing numbers of affected patients are being detected due to easy access to laboratory tests, more and more patients are likely to be subjected to surgical treatment, especially when it can be done as a simple outpatient procedure.

In this study we have evaluated the role of high-resolution USG in the preoperative localization of abnormal parathyroid glands and compared it to Tc-99m scintigraphy and findings at surgery.

## Material and Methods

### Subjects

Twenty-five consecutive patients with a diagnosis of PHPT underwent cervical USG followed by scintigraphy (Tc-99m) from 2004 to 2006. The diagnosis of PHPT was established by finding elevated serum calcium, phosphorus, and parathyroid hormone (PTH) levels as measured by solid-phase immunoassay.

### USG

**Figure 1:**
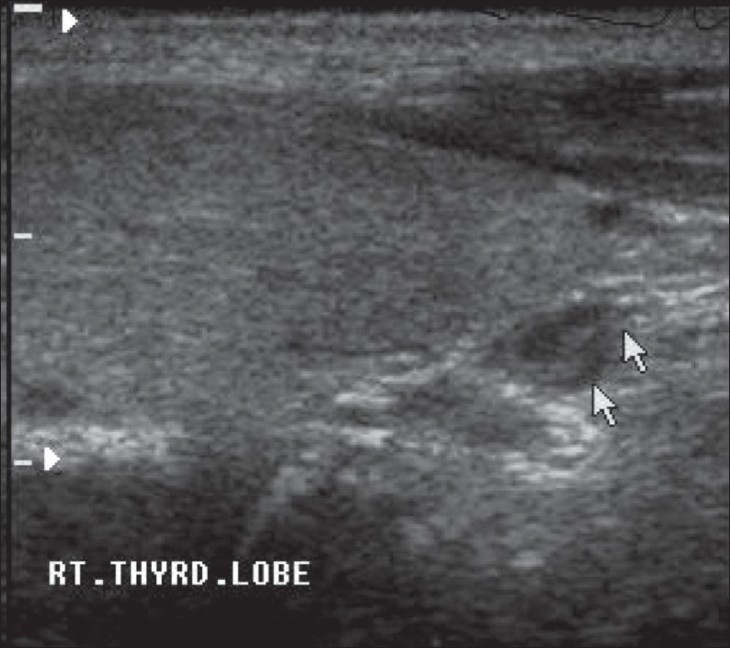
Longitudinal USG shows a parathyroid adenoma (arrow) inferior to the right lobe of the thyroid gland

**Figure 2:**
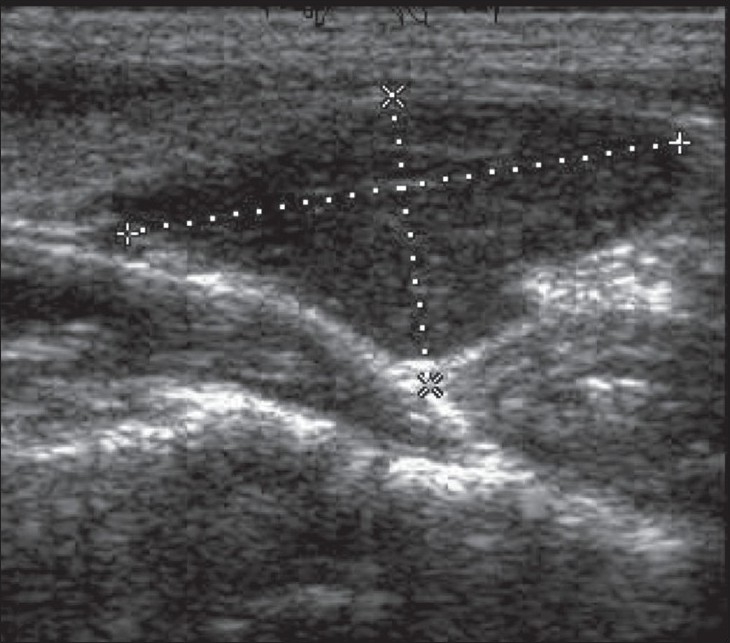
Longitudinal USG shows a large parathyroid adenoma inferior to the left thyroid lobe

USG was performed with a high-resolution linear transducer of 12 MHz frequency (ATL-HDI 1500, Philips). The examiner was blinded to the results of any previous imaging. USG was performed with the neck extended in the supine position, with the thyroid gland as the reference; scanning was done cephalad and caudad from the reference point up to the submandibular gland and the sternal notch, respectively. Parathyroid adenoma was recognized as a well-defined mass with a homogenous echo pattern, hypoechoic to thyroid tissue and with a fatty cleavage plane separating it from the adjacent thyroid lobe [Figure 1]. The gland position was defined as superior or inferior to the thyroid gland [Figure 2].

### Scintigraphy

Scintigraphy was performed by using technetium-99m sestamibi (Tc-99m) with early (20 min) and delayed (2 h) planar pinhole views of the neck and chest. Delayed (2–h) scans showing persistent retention of activity or areas with disproportionately increased sestamibi uptake compared to surrounding thyroid tissue were diagnosed as parathyroid adenomas.

### Surgery

All patients in our series had bilateral neck exploration under general anesthesia. Removal of the abnormal parathyroid tissue was confirmed by attaining > 50% reduction in intraoperative PTH levels 90 min after parathyroidectomy and by persistent normalization of serum calcium levels 6 months after surgery.

## Results

There were 25 patients in the study group, 17 men and 8 women, ranging in age from 18–55 years. Three patients presented with full-blown clinical features of hyperparathyroidism, including pathological fractures, renal calculi, and psychiatric symptoms; 16 patients presented with pathological fractures and renal calculi; and 6 patients with bilateral renal calculi.

The values of the most recent biochemical tests were calcium: 12.2–15.5 mg/dl, serum phosphorus: 1.7–4.6 mg/dl, and serum PTH: 450–2400 ng/l.

There were 28 abnormal glands in these 25 patients: 22 solitary and 6 multiple (two each in three patients). All the patients had curative surgery, which was defined as an intraoperative iPTH < 50% of the preoperative level at 90 min after parathyroid resection and a persistently normal postoperative serum calcium and iPTH at 6 months after surgery. Histopathology revealed chief- or clear-cell adenomas; there was one case of hyperplasia and one of carcinoma, both of which could not be diagnosed on USG or scintigraphy and were reported as adenomas. USG localized the adenomas in 20 out of 22 patients with single parathyroid adenomas and could localize 4 out of 6 adenomas in the three patients with multiple parathyroid adenomas. All positive lesions on USG were found at the correct side and site. The detection rate was 95% in glands weighing > 4000 mg and approximately 40% in glands weighing < 1500 mg. The location of an enlarged gland was classified as superior, inferior, or ectopic.

Scintigraphy detected 21 out of 22 solitary parathyroid adenomas and five of the six multiple adenomas in three patients. The correct side and site was established in all patients.

USG revealed a sensitivity of 91% and a positive predictive value (PPV) of 100% in solitary adenomas. Scintigraphy had a sensitivity of 95% and a PPV of 100% in the detection of solitary adenomas. In patients with multiple adenomas, USG had a sensitivity of 66% and a PPV of 100%, while Tc-99m sestamibi scintigraphy had a sensitivity of 83% and a PPV of 100%. Overall detection rates were 24 out of 28 adenomas (sensitivity 85.7%) for USG and 26 out of 28 adenomas (sensitivity 92.9%) for scintigraphy. Of the four adenomas missed on USG two were located in mediastinum and two in the lower neck. The two adenomas missed on scintigraphy were located in the mediastinum [Tables 1–2].

**Table 1:** Results of USG and Tc-99m sestamibi scintigraphy in patients with solitary adenoma

Imaging result	USG	Tc-99m sestamibi
True positive	20/22 (91)	21/22 (95)
False negative	2/22 (9.5)	1/22 (4.5)
Sensitivity	20/22 (91)	21/22 (95)
PPV	20/20 (100)	21/21 (100)

Figures in parentheses are in percentage

**Table 2:** Results of USG and Tc-99m sestamibi scintigraphy in patients with multiple adenomas

Imaging result	USG	Tc-99m sestamibi
True positive	4/6 (66)	5/6 (83)
False negative	2/6 (33)	1/6 (17)
Sensitivity	4/6 (66)	5/6 (83)
PPV	4/4 (100)	5/5 (100)

Figures in parentheses are in percentage

## Discussion

PHPT has an approximate incidence of 28 cases per 100,000 persons per year. A solitary adenoma is the commonest cause of hyperparathyroidism, accounting for 85–90% of patients; the other causes being hyperplasia (12–15%), multiple adenomas (2–3%), and parathyroid carcinoma (< 1%).[1,2] Over the years, there has been a growing trend towards focused operative removal of parathyroid adenomas using minimally invasive methods. The success of these methods is borne out by the findings of a large number of studies.[5–10] Even as far back as 1982, it was shown that unilateral neck exploration using preoperative localization would significantly reduce operative time compared to bilateral exploration and it was suggested then that the policy of bilateral neck exploration be eliminated.[11] One prospective randomized controlled study involving 91 patients showed that patients undergoing a unilateral procedure had a lower incidence of biochemical and severe symptomatic hypocalcemia in the early postoperative period compared with patients undergoing bilateral exploration.[10] Some authors even advocate outpatient minimally invasive parathyroid resection using cervical block anesthesia in selected patients in whom there is evidence of a solitary enlarged parathyroid gland on USG or scintigraphy.[5] A preoperatively localized adenoma can be conveniently excised by performing a limited neck dissection,[11] which requires shorter operative time and has lower complication rates.[12] Precise preoperative localization is thus a prerequisite. The different methods of preoperative localization vary in their accuracy, eg, USG: 70%, CT scan: 67%, MRI: 73%, MIBI scintigraphy: 78%.[2] Out of these, USG and scintigraphy are the two most commonly used and convenient modalities.

Scintigraphy is slightly more accurate than USG in detecting parathyroid adenomas, as it is capable of locating ectopic glands that are inaccessible by USG. In one study,[13] among patients undergoing both tests, Tc-99m scintigraphy was more likely than USG to yield a positive test result (92% *vs* 80%). The likelihood of correctly predicting the surgical results was not significantly different between the two methods (74% for USG and 82% for scintigraphy) and the PPV of the two tests was almost similar (90% *vs* 93% for USG and scintigraphy, respectively). The slightly greater likelihood of a positive test with scintigraphy was attributable to its higher sensitivity in detecting ectopic adenomas and in identifying cases of multigland disease.

For non-ectopic solitary adenomas, there was no significant difference in the likelihood of a correct positive test (85% for USG *vs* 82% for scintigraphy). Thus, USG could be used as first-line investigation for parathyroid adenoma. Since the main reason for USG missing an adenoma is ectopic location in the mediastinum or lower neck, scintigraphy is a good second-line investigation in cases where USG is negative. This approach should result in accurate preoperative localization in the majority of patients.

In our series of 25 patients, USG successfully located parathyroid adenomas in 24 out of 28 adenomas (20 out of 22 solitary adenomas and 4 out of 6 multiple adenomas). Neither USG nor scintigraphy could differentiate adenoma from hyperplasia or carcinoma, all being reported as adenoma. Thus, had we followed the policy of unilateral parathyroid resection, USG would have ensured success in 24 out of 28 adenomas, and in the two negative cases, the addition of scintigraphy would have ensured success in 26 adenomas out of 28. Given the ease of performance, ready availability, and its noninvasive nature, USG makes an robust, low-cost, and accurate modality for preoperative localization of parathyroid adenomas. In a series of 46 patients, Bhansali *et al.* reported that USG correctly localized abnormal parathyroid glands in 73%, whereas scintigraphy was positive in 98%. They also found that the reliability of positive findings by the two methods was similar in single adenomas, with a PPV of 100% for both USG and scintigraphy based on correlation with surgical findings.[14]

Since USG is being widely used for the localization of parathyroid adenomas, its potential usefulness in curative ablation of such adenomas is also being increasingly recognized. USG-guided ethanol ablation resulted in successful treatment in 11 out of 14 patients with parathyroid adenomas who were followed for a median of 39 months.[15] USG can thus be safely put to use in ethanol or laser ablation.[16]

MRI and CT scan have been used sparingly in parathyroid detection due to their high cost and reduced availability as compared to USG. However, in some specific situations MRI especially offers better soft tissue resolution. In one study on patients with PHPT, MRI was able to successfully locate all ectopic parathyroid adenomas[17] CT scan has also been found useful, but both CT scan and MRI are not as quick and easy to perform as USG.

The use of intraoperative parathyroid assay is very helpful in determining the success of surgery, especially in minimally invasive and focused surgical approaches. In one series, focused surgery was extended to bilateral exploration when intraoperative PTH failed to decrease by more than 50% relative to the highest baseline level.[18] Intraoperative PTH assessment was part of the surgical protocol in our series of bilateral neck exploration. Its use in limited neck explorations is obvious, as it determines the success of the procedure.

## Conclusion

With unilateral, focused, and minimally invasive surgical methods of parathyroidectomy coming into vogue, surgical treatment of PHPT can be offered to patients outside the tertiary hospital setting at a low cost and with negligible complications. USG, as an accurate preoperative localization modality, should thus become the first-line investigation. With proper training of sonologists, parathyroid detection should no longer be difficult and should, in future, result in more and more patients of PHPT being treated in non-tertiary care hospitals. A further useful application of USG would be in the percutaneous therapeutic ablation of adenomas, particularly in seriously ill patients.

## References

[1] Norton JA, Brenham MF, Wells S, Bilazikian JP, Marcus R, Levine MA Surgical management of hyperparathyroidism. The Parathyriods, Basic and Clinical Concepts.

[2] Kazya S, Koichiro A, Kazuo M, Ken-Ichi M (2003). Usefulness of diagnostic imaging in PHPT. Int J Urol.

[3] Inabnet WB, Fulla Y, Richard B, Bonnichon P, Icard P, Chapuis Y (1999). Unilateral neck exploration under local anesthesia: The approach of choice for asymptomatic PHPT. Surgery.

[4] Pelletteri PK (2003). Directed parathyroid exploration and evaluation of this approach in a single institution review of 346 patients. Laryngoscope.

[5] Casara D, Rubello D, Pelizzo MR, Shapiro B (2001). Clinical role of 99m TcO4/MIBI scan, ultrasound and intra-operative gamma probe in the performance of unilateral and minimally invasive surgery in PHPT. Eur J Nucl Med.

[6] Cougard P, Goudet P, Bilosi M, Peschaud F (2001). 2001 Videoendoscopic approach for parathyroid adenomas: Results of a prospective study of 100 patients (in French). Ann Chir.

[7] Quiros RM, Alioto J, Wilhelm SM, Ali A, Prinz RA (2004). An algorithm to maximize use of minimally invasive parathyroidectomy. Arch Surg.

[8] Jacobson SR, van Heerden JA, Farley DR, Grant CS, Grant CS, Thompson GB (2004). Focused cervical exploration for PHPT without intraoperative parathyroid hormone monitoring or use of gamma probe. World J Surg.

[9] Chen H, Sokoll LJ, Udelsman R (1999). Outpatient minimally invasive parathyroidectomy: A combination of Sestambi-SPECT localization, cervical block anaesthesia and intraoperative parathyroid harmone assay. Surgery.

[10] Bergenfelz A, Lindblom P, Tibblin S, Westerdahl J (2002). Unilateral versus bilateral neck exploration for PHPT: A prospective randomized control trial. Ann Surg.

[11] Tibbli S, Bondenson AG, Ljungberg O (1982). Unilateral parathyroidectomy in hyperparathyroidism due to single adenoma. Ann Surg.

[12] Fine EJ (1987). Parathyroid imaging: Its current status and future role. Semin Nucl Med.

[13] Wei JP, Burke GJ (1995). Analysis of savings in operative time for PHPT using localization with technetium 99 m sestambi scan. Am J Surg.

[14] Haber RS, Kim CK, Inabnet WB (2002). Ultrasonography for pre-operative localization enlarged parathyroid glands in PHPT: Comparison with Tc99 scintigraphy. Clin Endocrinol.

[15] Bhansali A, Masoodi SR, Bhadada S, Mittal BR, Behra A, Singh P (2006). Ultrasonography in detection of single and multiple abnormal parathyroid glands in PHPT: Comparison with radionuclide scintigraphy and surgery. Clin Endocrin (Oxf).

[16] Karstrup S, Hegedus L, Holm HH (1993). Ultrasonically guided chemical parathyroidectomy in patients with PHPT: A follow up study. Clin Endocrinol (Oxf).

[17] Adamek S, Libansky P, Nanka O, Sedy J, Pafko P (2005). Surgical therapy in PHPT and its complications. Zentralbl Chir.

[18] Haciyanli M, Lal G, Morita E, Duh QY, Kebebew F, Clark OH (2003). Accuracy of pre-operative localization studies and intra-operative parathormone assay in patients with PHPT and double adenoma. J Am Coll Surg.

